# The outbreak of the 2022 Russo-Ukrainian war: mental health of poles and their attitude to refugees

**DOI:** 10.3389/fpubh.2023.1155904

**Published:** 2023-06-23

**Authors:** Mateusz Babicki, Krzysztof Kowalski, Agnieszka Mastalerz-Migas

**Affiliations:** ^1^Department of Family Medicine, Wroclaw Medical University, Wrocław, Poland; ^2^Department and Clinic of Psychiatry, Wroclaw Medical University, Wrocław, Poland

**Keywords:** refugees, mental health, attitudes toward refugees, GHQ-28, anxiety

## Abstract

**Introduction:**

The outbreak of the Russo-Ukrainian war on 24 February 2022 has sparked a migration crisis in Europe. As a result, Poland has emerged as the country with the highest number of refugees. Due to differing social and political sentiments, this has been a significant challenge for the hitherto mono-ethnic Polish society.

**Methods:**

Computer-assisted web interviews (CAWIs) were conducted with 505 Poles, mainly women with higher education from large urban centers, involved in helping refugees. Their attitudes toward refugees were assessed using an original questionnaire, while their mental health was also evaluated using the General Health Questionnaire-28 (GHQ-28).

**Results:**

The vast majority of respondents reported favorable attitudes toward refugees from Ukraine. In addition, 79.2% believed refugees should be given free access to medical care, and 85% supported free access to education for migrants. Nearly 60% of respondents were not worried about their financial status due to the crisis; moreover, 40% believed that immigrants could boost the Polish economy. And 64% believed it would enrich Poland culturally. However, the majority of respondents feared infectious diseases and believed migrants should be vaccinated according to the vaccination schedule applicable in the country. Fear of war correlated positively with fear of refugees. On the GHQ-28, almost half of the respondents scored above clinical significance. Higher scores were typical for women and those fearing war and refugees.

**Conclusion:**

Polish society has shown a tolerant attitude in the face of the migration crisis. The vast majority of respondents showed positive attitudes toward refugees from Ukraine. The ongoing war in Ukraine has a negative impact on the mental health of Poles, which correlates with their attitude toward refugees.

## 1. Introduction

24 February 2022 will go down in the history of the world. It was the day Russian troops entered Ukraine and the largest armed conflict in Europe since the end of the Second World War began (1). War is undoubtedly a drastic experience that contributes to dramatic changes in the daily lives of the population. The ongoing conflict has many political, economic or health consequences (2). It is also the cause of the biggest migration crisis Europe has faced in the 21st century (3). According to data, more than six million Ukrainians have left their country and are seeking safety outside its borders (4). By far, the most common migration destination is Poland. In the first 30 days of the invasion, nearly 2.5 million Ukrainians arrived in Poland, the vast majority of whom may stay longer (5). By comparison, Europe’s largest migration crisis to date, between 2015 and 2016, involved 476,510 refugees from the Middle East settling in Germany (6). This situation is undoubtedly a massive challenge for the whole country, which has hitherto been relatively ethnically homogeneous and reluctant to admit refugees (7). Poles had to quickly adapt to a new reality and share their country with their closest neighbor. This state of affairs arguably stirs many emotions and concerns, often extreme ones. According to earlier reports, the attitudes of Poles toward both war and religious refugees were not favorable (8). The vast majority of Poles were reluctant to speak out for admitting them to the country, and the situation was accompanied by intense political debate. The situation was also aggravated by biased media content showing refugees in a bad light (9). In a 2021 the ongoing refugee crisis on the Polish-Belarusian border brought about widespread unease and posed a danger. In one research 48% of respondents did not want them to be let into the country (10). Government decisions at the time were extremely different from today’s, making migration difficult. Ultimately it resulted in the construction of a wall guarded by services. However, this did not stop the public from organizing grassroots underground support, including medical support for refugees (11). Given the scale of the phenomenon and public discord over the government’s migration policy, nowadays the situation is unique in the history of modern Poland. It proved to be a test of sorts for Polish civil society requiring the ability to manage the migration crisis (12).

Moreover, the ongoing armed conflict just outside the country’s borders is undoubtedly not conducive to mental health. As we know from previous reports, war has a devastating effect on mental health (13). Research has shown that the risk of developing depression, anxiety or post-traumatic stress disorder (PTSD) increases with ongoing conflict (14). Children appear to be a particular group of affected individuals (15). Furthermore, Polish society appears to suffer from mental fatigue in response to the ongoing COVID-19 pandemic and the waves of disease that followed almost one after the other, as manifested by significantly higher rates of anxiety and depression compared to the pre-pandemic status. Simultaneously, the quality of life became lower due to economic burden. Such effects were more pronounced in women and residents of big cities. (16).

In response to the ongoing problem, the Polish government has introduced a number of measures for Ukrainian refugees, such as easier access to work, social benefits and free medical care, including vaccinations against COVID-19 (17).

Therefore, this study aims to assess the impact of the ongoing war on the mental health of Poles. In addition, it evaluates public attitudes toward refugees from Ukraine. To the best of the authors’ knowledge, this is the first study of this kind conducted in Poland and worldwide, which is a testament to its innovation and strength.

## 2. Methodology

### 2.1. Methods

The present survey was based on an original questionnaire distributed online *via* a social media site (Facebook.com). The information about the survey was distributed in groups with a variety of topics in order to reduce the risk of sampling error. The distribution period of the survey was 20.03–07.04.2022, i.e., during the first two months of the outbreak of the war, when the largest influx of refugees was observed in Poland (5). It was addressed to all Poles aged 18 and over with Internet access. Exclusion criteria included lack of consent to participate in the study and age under 18. The survey was voluntary and anonymous, and respondents could quit it at any stage. Before their participation, the respondents were informed about the research methodology and objectives, after which informed consent was obtained from those willing to participate.

The survey was approved by the Ethics Committee of Wroclaw Medical University and conducted in accordance with the Declaration of Helsinki.

The original questionnaire consisted of three parts. The first included questions evaluating socio-demographic status: age, sex, place of residence, level of education, occupation or having children. The next part of the questionnaire concerned the evaluation of the attitudes of Poles toward refugees from Ukraine. It employed original questions based on a 5-point Likert scale, with the following responses: 1 – Strongly disagree; 2 – Disagree; 3 – Neither agree nor disagree; 4 – Agree; 5 – Strongly agree. Questions covered aspects of Poland’s reception of refugees, the availability of medical care, schooling or social benefits. In addition, respondents were also asked for their opinion on the impact of refugees on the labor market, economic development and cultural richness. Respondents were also asked whether they feared the refugees, and whether they were concerned about an increase in infections with diseases such as measles and polio, and whether refugees should be vaccinated according to the Polish vaccination schedule. The next part included questions based on a 10-point Likert scale assessing fear of refugees and fear of war as well as the likelihood of accepting a refugee into one’s home.

The final stage of the study involved a standardized psychometric tool, the GHQ-28. As the name suggests, it consists of 28 questions and is commonly used to assess psychological disorders. It is based on a 4-point Likert scale. The maximum score possible is 84 points (18). In addition, a score of 24 was considered a clinically significant cut-off point. Apart from the total score, the analysis can also include the tool’s assessment subscales:

- Somatic symptoms (items 1, 3, 4, 8, 12, 14, and 16)
- Anxiety and insomnia (items 2, 7, 9, 13, 15, 17, and 18)
- Social dysfunction (items 5, 10, 11, 25, 26, 27, and 28)
- Depression (items 6, 19, 20, 21, 22, 23, and 24)

### 2.2. Statistical analysis

Variables were of qualitative and quantitative nature. Basic descriptive statistics were used in the analysis of quantitative variables to compare the groups. Simple linear models were used to compare quantitative variables. The dependent variable comprised the total score of the GHQ-28 scale and its individual subscales. Independent variables included age, gender, place of residence, level of education, having children and fear of war, fear of refugees and assessment of Poland’s continued acceptance of refugees. In contrast, comparisons of qualitative variables were made using a chi-squared test. Correlation between quantitative variables was assessed using the Spearman correlation test. The statistical significance level was established at *p* < 0.05 for each case. Calculations were performed using Statistica 13.0.

## 3. Results

### 3.1. Characteristics of the study group

The survey involved 505 respondents from all over Poland with 100% consent to participate. The study group comprised mainly women – 408 (80.8%), and people living in large cities – 228 (45.1%). The mean age was 32.7 ± 9.6 years. The study group included 184 (36.4%) respondents working as healthcare professionals and 211 (41.8%) having at least one child. A detailed summary is shown in Table 1.

**Table 1:** Characteristics of the study group.

Variable		N/M	%/SD
Age		32.7	9.61
Sex	Female	408	80.8
	Male	97	19.2
Place of residence	City of over 250,000 inhabitants	228	45.1
	City of 50,000–250,000 inhabitants	98	19.4
	Town of up to 50,000 inhabitants	86	17.0
	Rural area	93	18.5
Part of Poland	East	169	33.5
	West	336	66.5
Level of education	Higher (university degree)	319	63.2
	Other	186	36.8
Marital status	Married	220	43.5
	Partnership	170	33.7
	Single	115	22.8
Children	Yes	211	41.8
	No	294	58.2
Health professional	Yes	184	36.4
	No	321	63.6

N, number; M, mean; SD, standard deviation.

### 3.2. Attitude toward refugees from Ukraine in the face of an ongoing war

The vast majority of respondents showed favorable attitudes toward refugees from Ukraine and agreed that Poland should remain open to refugees. Furthermore, 79.2% of respondents believed that refugees should be provided with free access to medical care and the opportunity to receive education (85%). Furthermore, nearly 60% of respondents did not fear a reduction in earning opportunities due to the influx of refugees, while 40% believed that the influx of refugees would boost the country’s economy, and 64% thought the country would also be culturally enriched.

However, more than half of respondents feared that the influx of refugees could lead to local outbreaks of infectious diseases such as polio and measles. More than 80% of respondents thought that both children and adults should be vaccinated according to the applicable vaccination schedule in Poland. Less than 30% of respondents expressed concern that the level of security in Poland would decrease with the influx of refugees. A detailed summary of questions is shown in Table 2.

**Table 2:** Summary of questions assessing Poles’ attitudes toward refugees from Ukraine.

	Strongly disagree	Disagree	Neither agree nor disagree	Agree	Strongly agree
	*N* (%)				
Should Poland accept refugees without restriction?	46 (9.1)	100 (19.8)	42 (8.3)	186 (36.9)	131 (25.9)
Refugees should receive free healthcare.	16 (3.2)	51 (10.1)	38 (7.5)	253 (50.1)	147 (29.1)
Should refugees receive social benefits?	34 (6.7)	126 (25.0)	76 (15.1)	186 (36.8)	83 (16.4)
Can refugees take jobs away from Poles?	67 (13.3)	238 (47.1)	68 (13.5)	105 (20.7)	27 (5.3)
Will the influx of refugees worsen financial conditions in the labor market?	44 (8.6)	207 (41.0)	64 (12.7)	131 (26.0)	59 (11.7)
Refugees can take full advantage of care and study opportunities in Poland	9 (1.8)	34 (6.7)	33 (6.5)	235 (46.5)	194 (38.5)
Can refugees contribute to infectious disease outbreaks (e.g., measles/polio)?	35 (6.9)	105 (20.8)	102 (20.2)	190 (37.6)	73 (14.5)
Should refugees be vaccinated as soon as possible in accordance with the vaccination schedule in Poland?	11 (2.2)	26 (5.1)	57 (11.3)	202 (40.0)	209 (41.4)
The influx of refugees is reducing the level of security in Poland?	57 (11.3)	240 (47.5)	71 (14.1)	98 (19.4)	39 (7.7)
Refugees will enrich the country culturally	20 (4.0)	62 (12.3)	99 (19.6)	232 (45.9)	92 (18.2)
The influx of refugees will contribute to Poland’s economic growth.	27 (5.3)	114 (22.6)	162 (32.1)	149 (29.5)	53 (10.5)

Looking at questions based on a 10-point Likert scale, respondents averaged a score of 2.97 ± 2.4 in assessing their fear of refugees, with the most common response being 1. For fear of war, it was 5.76 ± 2.68, with the most common response being 7. The scores for the above questions showed a weak positive correlation (*r* = 0.185; *p* < 0.001). When assessing the likelihood of welcoming a refugee into their home, respondents averaged a score of 4.97 ± 3.2, but the most common response was 1.

In the assessment of sociodemographic variables, it was shown that those with higher education showed more favorable attitudes both in their assessment of Poland’s continued acceptance of refugees, access to free medical care and social allowances. In addition, medical professionals were significantly more likely (91.3% vs. 75.7%, *p* < 0.001) to believe that refugees should undergo compulsory vaccinations in Poland. There were no differences between the inhabitants of the eastern and western parts of Poland with respect to the questions analyzed. Responses to individual questions on attitudes toward refugees by socio-demographic variables are presented in Table 3.

**Table 3:** Attitudes toward refugees in relation to socio-demographic variables.

Variable		Welcoming refugees		Free healthcare		Social benefits		Use of care and education services by refugee children		Refugees will contribute to economic growth		. The influx of refugees will reduce the level of insecurity in Poland		Mandatory vaccination according to the vaccination schedule in Poland	
		Percentage of people agreeing or strongly agreeing with a given statement													
		*N* (%)	*p*	*N* (%)	*p*	*N* (%)	*p*	*N* (%)	*p*	*N* (%)	*p*	*N* (%)	*p*	*N* (%)	*p*
Sex	Female	248 (60.7)	**0.003**	323 (79.2)	0.011	214 (52.5)	**0.027**	343 (84.1)	0.022	146 (35.7)	**<0.001**	114 (27.9)	0.048	336 (82.4)	0.537
	Male	39 (71.3)		77 (79.3)		55 (56.7)		86 (88.7)		56 (57.7)		23 (23.7)		75 (77.3)	
Place of residence	Rural area	55 (59.1)	0.867	75 (80.6)	0.434	47 (50.5)	0.383	78 (83.9)	0.588	34 (36.5)	0.522	30 (32.2)	0.481	70 (75.3)	**0.036**
	Town of up to 50,000 inhabitants	55 (63.9)		66 (76.7)		51 (59.3)		78 (90.7)		30 (34.8)		25 (29.1)		67 (76.9)	
	City of 50,000–250,000 inhabitants	58 (59.2)		74 (75.5)		47 (47.9)		83 (84.5)		36 (36.7)		28 (28.5)		75 (76.5)	
	City of over 250,000 inhabitants	149 (65.4)		185 (85.1)		124 (54.4)		190 (83.3)		102 (44.7)		54 (23.7)		199 (89.2)	
Level of education	Higher (university degree)	216 (67.7)	0.002	263 (82.4)	0.008	180 (56.4)	**0.009**	269 (84.3)	0.312	139 (43.6)	**0.239**	82 (25.7)	0.485	272 (85.3)	**0.017**
	Other	101 (54.3)		137 (73.6)		89 (47.8)		160 (86.0)		63 (33.8)		55 (29.6)		139 (74.3)	
Marital status	Married	140 (63.6)	0.143	171 (77.7)	0.292	122 (55.5)	**0.014**	189 (85.9)	0.194	94 (42.7)	0.321	52 (23.6)	0.074	189 (85.9)	0.522
	Partnership	100 (58.8)		133 (78.2)		76 (44.7)		138 (81.2)		54 (31.7)		57 (33.5)		1,356 (79.4)	
	Single	77 (66.9)		96 (83.4)		71 (61.7)		102 (88.7)		40 (34.7)		28 (24.3)		87 (75.6)	
Healthcare professional	Yes	130 (70.6)	**0.041**	185 (84.2)	**0.048**	109 (59.2)	0.258	154 (83.7)	0.219	76 (41.3)	0.963	44 (23.9)	0.298	168 (91.3)	**<0.001**
	No	187 (58.3)		245 (76.3)		160 (49.8)		275 (85.6)		126 (39.3)		93 (28.9)		243 (75.7)	
Children	Yes	137 (64.9)	0.656	167 (79.1)	0.839	124 (58.8)	0.104	180 (85.3)	0.132	92 (43.6)	0.203	54 (25.6)	0.395	177 (83.8)	0.367
	No	180 (61.2)		233 (79.3)		145 (49.3)		249 (84.7)		110 (37.4)		83 (23.2)		234 (79.5)	
Part of Poland	East	95 (56.2)	0.091	133 (78.7)	0.355	86 (50.8)	0.559	289 (86.0)	0.356	138 (41.1)	0.496	86 (25.6)	0.160	268 (79.7)	0.637
	West	222 (66.1)		267 (79.5)		183 (54.5)		140 (82.8)		64 (37.8)		51 (30.2)		143 (84.6)	

Significant effects (<0.05) are marked in bold.

### 3.3. The impact of war on mental health

In the analysis of the GHQ-28, the mean score of respondents was 25.03 ± 13.5 points, with 238 (47.1%) respondents scoring above clinical significance. The linear analysis showed no significant differences in both total scale or subscale scores for anxiety and somatic symptoms in relation to place of residence, level of education or relationship status. Women had an average score higher than men by 1.978. They also scored higher on individual subscales. There were no differences in the total GHQ-28 and subscale scores between the eastern and western parts of the country.

In a question assessing the fear of war in Poland, respondents averaged a score of 5.76 ± 2.68. The most common response was 6. Furthermore, linear models showed that as fear of war and refugees increases, the total GHQ-28 and subscale scores of anxiety and somatic symptoms increase. A detailed summary is shown in Table 4.

**Table 4:** The impact of sociodemographic variables and fear of refugees, willingness to accept refugees, and fear of war in Poland on the GHQ-28 scale and its individual subscales.

Variable		GHQ-28 positive		GHQ-28 total result				GHQ-28: Somatic symptoms				GHQ-28: Anxiety/Sleep disorder			
		%	*p*	Value	SD	*t*	*p*	Value	SD	*t*	*p*	Value	SD	*t*	*p*
Age		––	––	−0.07	0.062	−1.17	0.243	−0.001	0.018	−0.11	0.914	−0.013	0.022	−0.59	0.557
Sex	Female	50.3	**0.005**	1.978	0.758	2.60	**0.009**	0.599	0.217	2.76	**0.006**	1.055	0.269	3.92	**<0.001**
	Male	34.0		Ref.	Ref.	Ref.	Ref.	Ref.	Ref.	Ref.	Ref.	Ref.	Ref.	Ref.	Ref.
Place of residence	Rural area	46.2	0.116	−1.411	1.181	−1.19	0.234	−0.004	0.340	−0.14	0.887	−0.445	0.423	−1.05	0.293
	Town of up to 50,000 inhabitants	55.8		1.897	1.218	1.55	0.119	0.322	0.350	0.92	0.357	0.731	0.435	1.68	0.094
	City of 50,000–250,000 inhabitants	52.0		−0.475	1.163	−0.41	0.682	−0.217	0.334	−0.65	0.515	−0.158	0.412	−0.38	0.703
	City >250,00 inhabitants	42.1		Ref.	Ref.	Ref.	Ref.	Ref.	Ref.	Ref.	Ref.	Ref.	Ref.	Ref.	Ref.
Level of education	Higher (university degree)	46.1	0.856	−0.867	0.623	−1.39	0.164	−0.094	0.178	−0.52	0.600	−0.045	0.223	−0.201	0.841
	Other	46.4		Ref.	Ref.	Ref.	Ref.	Ref.	Ref.	Ref.	Ref.	Ref	Ref.	Ref.	Ref.
Marital status	Married	44.1	0.366	Ref.	Ref.	Ref.	Ref.	Ref.	Ref.	Ref.	Ref.	Ref	Ref.	Ref.	Ref.
	Partnership	47.7		−0.160	0.861	−0.18	0.853	−0.087	0.248	−0.35	0.725	−0.121	0.309	−0.39	0.697
	Singiel	52.2		1.784	0.855	1.86	**0.049**	0.189	0.275	0.69	0.490	0.263	0.343	0.77	0.443
Medics	Yes	43.5	0.249	Ref.	Ref.	Ref.	Ref.	Ref.	Ref.	Ref.	Ref.	Ref.	Ref.	Ref.	Ref.
	No	49.2		1.005	0.623	1.61	0.107	0.113	0.179	0.63	0.526	0.189	0.223	0.84	0.397
Children	Yes	42.7	0.123	Ref.	Ref.	Ref.	Ref.	Ref.	Ref.	Ref.	Ref.	Ref.	Ref.	Ref.	Ref.
	No	50.3		1.49	0.606	2.46	**0.014**	0.221	0.174	1.27	0.204	0.206	0.218	0.95	0.343
Region of Poland	East	51.5	0.165	Ref.	Ref.	Ref.	Ref.	Ref.	Ref.	Ref.	Ref.	Ref.	Ref.	Ref.	Ref.
	West	44.9		−0.953	0.636	−1.49	0.134	−0.243	0.183	−1.33	0.184	−0.414	0.223	−1.83	0.069
Fear of refugees		––	––	0.991	0.246	4.031	**<0.001**	0.226	0.071	3.186	**0.002**	0.267	0.089	3.016	**0.003**
Willingness to host refugees		––	––	−0,273	0,199	−1,37	0.171	−0.012	0.052	−0.209	0.834	−0.112	0.071	−0.163	0.871
Fear of the outbreak of war in Poland		––	––	1.442	0.215	6.70	**0.001**	0.398	0.061	6.42	**0.001**	0.638	0.007	8.49	**0.001**

Significant effects (<0.05) are marked in bold.

## 4. Discussion

The study aimed to assess the attitudes of Poles toward refugees from Ukraine during the Russo-Ukrainian war and evaluate the impact of the warfare on the psychological well-being of Poles. This analysis shows that Poles demonstrate a high level of acceptance of refugees from Ukraine. The vast majority believe Poland should continue allowing refugees to enter the country. Furthermore, according to the respondents, these people should be provided with social support. They should also be allowed to start working as soon as possible, and their children should receive an education. Moreover, respondents believe that these people should also be provided with free healthcare. The provision of adequate healthcare is crucial given that the vast majority of refugees are women, children and the older adult, and therefore their health profile may be diverse (19). As part of its assistance, the Polish government has introduced a law guaranteeing refugees the use of medical services on an equal footing with Polish citizens, including preventive medical examinations and vaccinations against COVID-19. Arguably, this will pose a considerable challenge for the entire health system. On the one hand, healthcare professionals have been overwhelmed with responsibilities in the almost consecutive two waves of the COVID-19 pandemic and the upcoming ones (20). On the other hand, the financial burden, where, according to preliminary estimates by the Polish Minister of Health, the cost of providing full medical care will amount to PLN 300 million (approximately USD 68 million) for every million refugees (21). It should be mentioned that Poland’s healthcare system is one of the least funded in the European Union, with expenditure in 2015 amounting to 6.3% of GDP while the EU average was 9.9%. In addition, out-of-pocket spending on medical treatment is high at 22% (22). There is also a significant shortage of health professionals in the health system: doctors, nurses or paramedics (23). For instance, there are 10.2 psychiatrists per 100,000 in Poland. people, which is twice as low as the EU average (24). The incoming refugees have offered some hope for a solution to this problem. To this end, the Polish government has decided to lift restrictions on the employment of non-EU healthcare professionals, including the need for recognition of diplomas or Polish language examinations. However, according to the Ministry of Health, fewer than 1,000 doctors from Ukraine were recruited in the first three months after the war outbreak. The difficulty in recruiting more migrant doctors from Ukraine was due to the language barrier (25). At the same time, the number of independent volunteer initiatives to organize medical assistance for refugees by Polish residents and doctors is growing (26).

The health aspect also emerged as one of the main fears among respondents. More than 50% feared an increased risk of transmission of infectious diseases such as polio or measles with the influx of refugees. This can be attributed to the low level of vaccination against infectious diseases among Ukrainians (27). In the past, this has led to local outbreaks of measles and polio in Ukraine (28, 29). This is particularly important given the recent increase in vaccination evasion by Polish parents (30). In addition, HIV and TB incidence rates are significantly higher in Poland and the rest of the European Union, making it necessary to develop coherent strategies to counter the spread of these diseases, including screening and supporting the patients (31, 32). Therefore, more than 80% of respondents believed that people arriving in Poland should be vaccinated as soon as possible in accordance with the applicable vaccination schedule in Poland. However, by law, mandatory vaccination applies only to those residing in Poland for at least three months; otherwise, it is only recommended (33). Moreover, the rapid increase in the number of refugees presents more difficulties for the Polish healthcare system, which was already struggling with the effects of the COVID-19 outbreak and a lack of medical personnel (34).

In recent years, a significant number of economic migrants from Ukraine have arrived in Poland. Before the pandemic outbreak, there were 1.3 million Ukrainian nationals in Poland (35). Due to the similar cultural background, the assimilation of this population proceeded without significant disruption despite a few problems resulting from xenophobic behavior by Poles, illegal work and minor offenses by migrants (36). Hence, it is worth mentioning the “contact theory” by Allport, describing the fact that a positive attitude toward an immigrant population emerges when it is not unknown and alien (37). This is the case in the situation described in the study, which can positively affect attitudes toward war refugees. The political narrative also plays an important role. Today it is recognized that right-wing parties are hostile to refugees (38). Although Poland is ruled by the conservative party “Law and Justice,” the image of refugees from Ukraine is positive in the public media. This is due to solidarity toward a neighboring country in the face of Russian aggression (39). Psychologists stress that in emergencies, people are more inclined to help as a social norm, which may overlap synergistically in this case by the effect of collective resilience to danger (40). In addition, like Ukraine, Poland has historically been politically dependent and oppressed by Russia on many occasions (41).

Another critical factor in the acceptance of refugees is the present economic situation. More than half of the respondents were not worried that the influx of refugees would worsen the labor market in Poland; moreover, 40% thought that they would contribute to Poland’s economic growth (42). The influx of workers can increase the country’s economic potential (43). In addition, getting refugees into work early on would contribute to their independence and relieve the burden on the state budget. Monitoring this is extremely important because, as we know from previous observations, fear of economic stability can cause social discontent and increase resentment toward other groups (44). Also, our survey revealed that the respondents’ enthusiasm toward social benefits was much lower than the right to medical care or education. Meanwhile, upon being assigned a PESEL number, Ukrainian refugees became eligible for a number of social benefits enjoyed by Polish citizens and a one-off welfare payment of PLN 300 (approximately USD 60) (45).

At present, the literature contains scant data on the attitudes of Poles and other nations toward refugees from Ukraine in the face of the ongoing war. Based on previous opinion polls, we know that Poles showed varying levels of acceptance toward refugees from countries at war. During the migration crisis of 2015, as many as 72% of Poles thought refugees should be allowed to enter the country, 14% of whom also thought they should be allowed to stay in Poland. Over the years, there has been an increase in resentment, where in 2017, the acceptance level dropped to 35%. Another survey was conducted in 2021 when Poland faced a crisis on the border with Belarus. It stemmed from an influx of refugees from the Middle East who were trying to enter Europe *via* the Polish-Belarusian border. A total of 42% were in favor of allowing them into the country, of which 33% only for the duration of the conflict (10). In contrast, in a 2021 survey by the United Nations High Commissioner for Refugees, 77% of Poles said Poland should support refugees fleeing war. In the same survey, 62% of respondents believed Poland should take in war refugees (46). One preprint study showed sympathy toward refugees increased during the Ukraine crisis in ten developed countries. The authors caution, however, that this effect may be reduced as the number of refugees arriving increases, and at this point, those countries have accepted far fewer refugees than Poland (47).

Our analysis also showed that individual socio-demographic factors selectively influenced some attitudes toward refugees. Women showed a more favorable attitude toward refugees, which is in line with the results of an Australian study in which men, in particular, reported a higher degree of threat by refugees. It can be hypothesized that men may view refugees as a more significant resource threat than women, as a result of traditional gender stereotypes which assign men the role of being responsible for economic resources, safety, and community cohesion (48). Residents of large cities also showed less concern about the influx of refugees, which may be related to multiculturalism in large agglomerations (49). Highly educated respondents responded similarly, which is associated with a greater knowledge of foreign cultures, the ability to think critically and also indirectly with a higher salary (50). These factors are widely recognized as key to a positive perception of refugees (51). On the other hand, recent qualitative research from Poland has confirmed that Poles form small cities and countryside are more willing to reveal xenophobic attitude associated with the feeling of social injustice (52). Additionally, the image of Polish society in this category may be distorted by biased media reports (53).

Another aspect addressed in our survey is the mental health of Poles in response to the ongoing armed conflict. Unfortunately, no data on the impact of war on the psychological well-being of neighboring countries is available for comparison. In our study, the mean score of respondents on the GHQ-28 was 25, which was above clinical relevance, achieved by 47.1% of respondents. Women and those living alone were more likely to experience psychological strain in the face of war, which is also confirmed by data from global conflicts (54). Notably, those with a greater fear of war and refugees simultaneously had higher GHQ scores. Interestingly and worryingly, another study on the Polish population showed a positive relationship between fear of war and willingness to help (55).

The authors are aware of the limitations of this study, which is undoubtedly the CAWI data collection methodology. Using this method, the authors have no way of verifying the identity of the person or the veracity of the data provided. On the other hand, research shows that completing questionnaires online is associated with greater acceptance and likelihood of response (56, 57). Respondents are also more willing to express a genuine opinion, avoiding social pressure and giving more socially acceptable answers. Which is particularly relevant to the issue we are analyzing. The second limitation is that the study group is not representative of Polish society. The overwhelming predominance of women, residents of large cities, those with a university education, and medical professionals may have influenced the final results of the survey. Furthermore, the authors have no way of verifying the number of people reached by the survey and the response rate. It should be stressed that the survey only covered the most difficult period immediately after the outbreak of war, so the sentiments shown are likely to evolve over time. It should also be mentioned that due to the nature of the study and its anonymity, the authors are not in a position to communicate the results of the GHQ-28 scale and possible support for individual study participants. On the other hand, it is hoped that participation in the study will increase respondents’ awareness of their own mental health and that they will seek support when in doubt.

In contrast, the results of this study provide an important contribution to the assessment of public attitudes toward refugees in various social and economic aspects. On this basis, it is possible to develop appropriate campaigns to raise awareness among Poles about refugee rights and obligations. In addition, continuous monitoring of the phenomenon of the level of acceptance of refugees will allow early detection of possible social tensions.

In summary, this survey results indicate a high level of acceptance of war refugees from Ukraine among respondents in Poland. An overwhelming majority of respondents believed these people should be provided with social and medical care and allowed to enter the workforce, which could improve the country’s economy. However, with the influx of refugees, there are also fears of epidemiological risks arising from low vaccination rates. Social acceptance of refugees needs to be continuously monitored due to the protracted nature of the war. Moreover, the qualitative data in the topic of this research should be considered cautiously due to the high level of bias based on political worldview.

## 5. Conclusion

Polish society has shown an empathetic and humanitarian attitude in the face of the crisis. The vast majority of respondents showed positive attitudes toward refugees from Ukraine. Women and those better educated and from larger urban areas show a more favorable attitude. Psychoeducational activities should be expanded, especially among men and people with lower education and from smaller towns and villages, to increase understanding and friendliness toward refugees. In order to increase the effectiveness of such actions, economic support for Polish minority groups most vulnerable to the global crisis related to war seems essential. The ongoing war in Ukraine has a negative impact on the mental health of Poles, which correlates with their attitude toward refugees. It is also necessary to conduct longitudinal studies on a representative group of Poles.

## Data availability statement

The raw data supporting the conclusions of this article will be made available by the authors, without undue reservation.

## Ethics statement

The studies involving human participants were reviewed and approved by the Ethics Committee of Wroclaw Medical University. The patients/participants provided their written informed consent to participate in this study.

## Author contributions

MB: conceptualization, methodology, and formal analysis. MB and KK: writing—original draft. MB, KK, and AM-M: writing—review and editing, acquisition, and visualization. MB and AM-M: funding. AM-M: investigation and supervision. All authors contributed to the article and approved the submitted version.

## Funding

This research was founded by the Wroclaw Medical University SUBZ.C290.23.069.

## Conflict of interest

The authors declare that the research was conducted in the absence of any commercial or financial relationships that could be construed as a potential conflict of interest.

## Publisher’s note

All claims expressed in this article are solely those of the authors and do not necessarily represent those of their affiliated organizations, or those of the publisher, the editors and the reviewers. Any product that may be evaluated in this article, or claim that may be made by its manufacturer, is not guaranteed or endorsed by the publisher.
